# Digitalization of a non-irradiated acute myeloid leukemia model

**DOI:** 10.1186/s12918-016-0308-x

**Published:** 2016-08-26

**Authors:** Rudong Li, Hui Cheng, Tao Cheng, Lei Liu

**Affiliations:** 1 Key Laboratory of Systems Biology, Institute of Biochemistry and Cell Biology, Shanghai Institutes for Biological Sciences, Chinese Academy of Sciences, Shanghai, 200031, China; 2 State Key Laboratory of Experimental Hematology, Institute of Hematology, Chinese Academy of Medical Sciences & Peking Union Medical College, Tianjin, 300020 China; 3 Shanghai Center for Bioinformatics Technology, Shanghai, 201203 China; 4 Institutes for Biomedical Sciences, Fudan University, Shanghai, 200031 China

**Keywords:** Non-irradiated AML model, Computational modeling leukemia, Hematopoietic suppression, Increased quiescence of HSC

## Abstract

**Background:**

Computer-aided, interdisciplinary researches for biomedicine have valuable prospects, as digitalization of experimental subjects provide opportunities for saving the economic costs of researches, as well as promoting the acquisition of knowledge. Acute myeloid leukemia (AML) is intensively studied over long periods of time. Till nowaday, most of the studies primarily focus on the leukemic cells rather than how normal hematopoietic cells are affected by the leukemic environment. Accordingly, the conventional animal models for AML are mostly myeloablated as leukemia can be induced with short latency and complete penetrance. Meanwhile, most previous computational models focus on modeling the leukemic cells but not the multi-tissue leukemic body resided by both leukemic and normal blood cells. Recently, a non-irradiated AML mouse model has been established; therefore, normal hematopoietic cells can be investigated during leukemia development. Experiments based on the non-irradiated animal model have monitored the kinetics of leukemic and (intact) hematopoietic cells in multiple tissues simultaneously; and thus a systematic computational model for the multi-tissue hematopoiesis under leukemia has become possible.

**Results:**

In the present work, we adopted the modeling methods in previous works, but aimed to model the tri-tissue (peripheral blood, spleen and bone marrow) dynamics of hematopoiesis under leukemia. The cell kinetics generated from the non-irradiated experimental model were used as the reference data for modeling. All mathematical formulas were systematically enumerated, and model parameters were estimated via numerical optimization. Multiple validations by additional experimental data were then conducted for the established computational model. In the results, we illustrated that the important fact of functional depression of hematopoietic stem/progenitor cells (HSC/HPC) in leukemic bone marrow (BM), which must require additional experiments to be established, could also be inferred from our computation model that utilized only the cell kinetics data as the input.

**Conclusion:**

The digitalized AML model established in the present work is effective for reconstructing the hematopoiesis under leukemia as well as simulating the hematopoietic response to leukemic cell expansion. Given the validity and efficiency, the model can be of potential utilities in future biomedical studies; additionally, the modeling method itself can be also applied elsewhere.

**Electronic supplementary material:**

The online version of this article (doi:10.1186/s12918-016-0308-x) contains supplementary material, which is available to authorized users.

## Background

Animal models are constantly built and utilized in medical researches as manipulable platforms for investigations of diseases. As self-evident, an appropriate experimental model is able to represent and reproduce the phenotypes of disease progression, thus promoting the studies of disease physiology as well as therapeutics. Experimental models of mice have been usually applied for researches of leukemia, especially the acute myeloid leukemia (AML). However, most of the leukemia models established/utilized in previous studies rely on protocols of pre-conditioning the recipients by myeloabative manipulations such as immunosuppressive agents, total body irradiation or xeno-transplantation [1–3]. Although these models are efficient in leukemia induction with short latency and complete penetrance [4, 5], obviously, leukemogenesis generally does not occur in myeloablated or immuno-deficient hosts albeit they are robust in terms of disease progression. For example, in a typically pre-conditioned model, leukemia is induced by irradiating the host via total body irradiation followed by transplantation of hematopoietic cells overexpressed some retrovirus-mediated oncogenic proteins (eg Notch1 and MLL-AF9). Such manipulations destroy the normal HSC/HPC populations as well as the immune system in the recipients; and moreover, they pose a significant bystander effect on the transplanted cells in the marrow [6]. Thus the resulted leukemic host will greatly deviate from the condition of native leukemia development; and therefore, the traditional models mainly benefit the researches for leukemic cells.

In recent studies, functional alterations of normal hematopoietic cells in the environment of leukemia has been highlighted [4, 7–10]. To study “normal” hematopoietic response to leukemia, the traditional models would be problematic as discussed above. Hence, the study should be performed with an un-manipulated leukemia model in which a normal recipient mouse without any pre-conditioning is induced to develop leukemia, sustaining a physiological setting more comparable to that of native leukemogenesis. To this end, a robust non-irradiated (non-myeloablated) mouse model for AML has been recently established, with a 100 % penetrance of leukemia development [11]. Thus intact normal blood cells without overt damage could be directly measured during leukemia progression. Motivated by the value of this experimental model, we have digitalized it in the present work; the constructed computational model is able to mimic the cell kinetics of hematopoiesis under AML. Thus foremost, our effort may be beneficial for saving experimental costs; and in turn, potentially facilitating further biomedical researches. Meanwhile, results attained by the computational model have reaffirmed the progressive suppression of hematopoiesis under leukemia, as well as the increased quiescence of HSCs in leukemic BM.

## Results

### Workflow

**Fig. 1:**
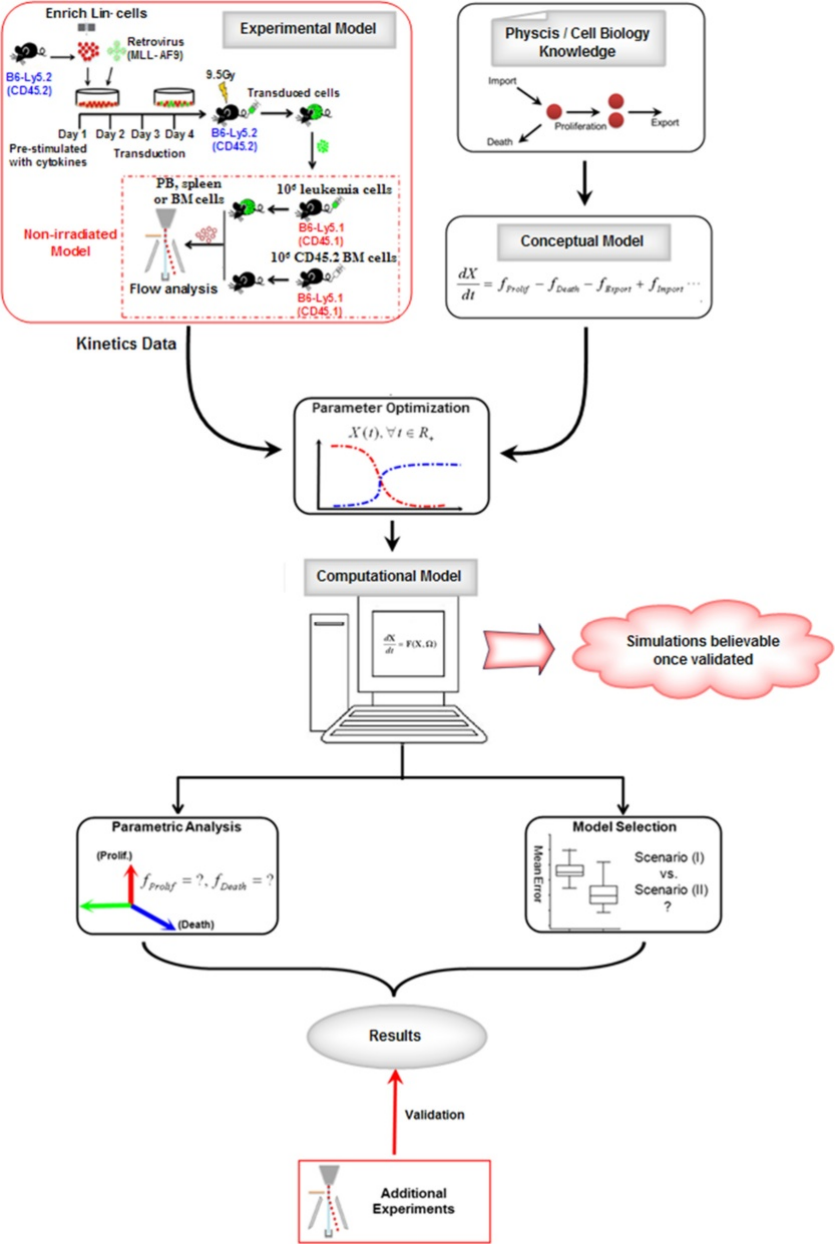
Modeling workflow. Adopting the cell kinetics measured from a non-irradiated AML mouse model, the computational model was established based on physics and biology knowledge via the procedures of parameter optimization (for details, see Methods). Using the model, numerical simulations could be implemented, eg parametric analysis and model selection. Furthermore, the model was validated with additional experimental results; and once the model validity was credited, the model could be of certain utilities

Raw data of cell kinetics were generated by a non-irradiated AML mouse model, in which normal recipient mice without any pre-conditioning were induced to develop leukemia at a 100 % penetrance [11]. In the meantime, we assembled the knowledge of physics and cell biology and then conceived a theoretical model (ie the conceptual model). Using the experimental data as reference, we derived a computational model through parameter optimization, which was the key step in the procedures of modeling. Aftermath, we conducted several in silico experiments based on the model mainly by means of parametric analysis and model selection, and then verified the computational results with additional experimental references. Once the model was validated, its predictions (or derivative deductions) could be regarded as be credible (Fig. 1).

### Parameter optimization

#### Model for the hematopoiesis dynamics of leukemic body

**Fig. 2:**
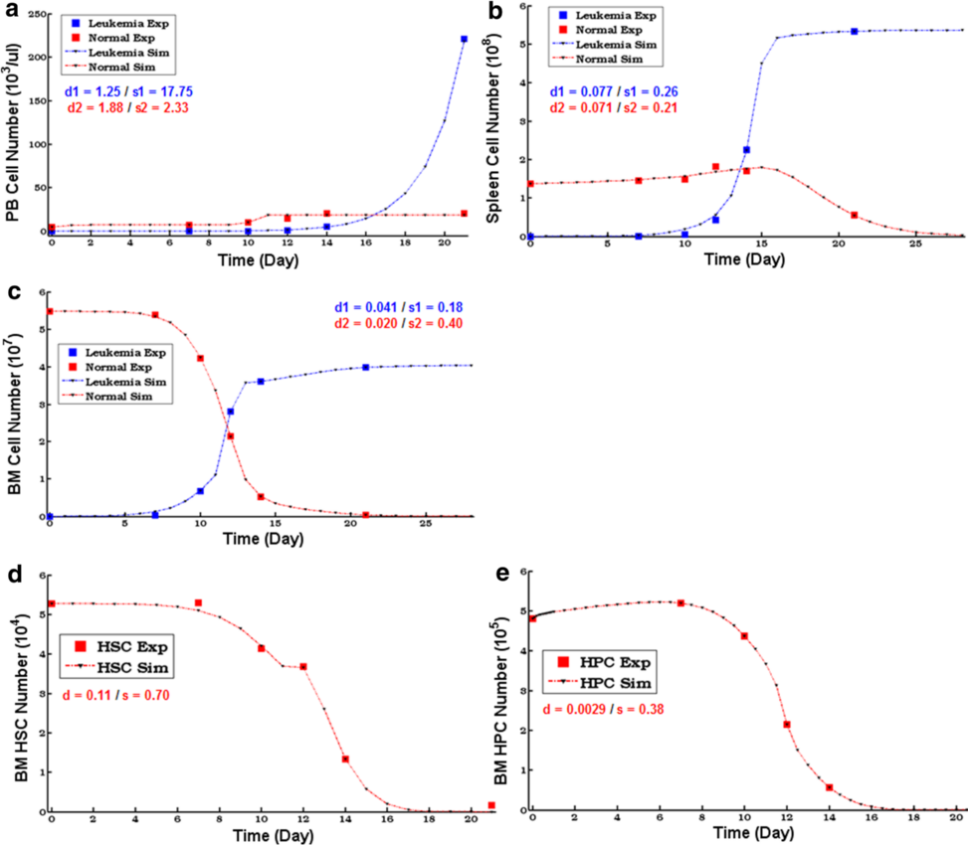
Cell kinetics simulations of the model with the optimized parameters. (**a**–**c**). Computational dynamics of the normal and leukemic cells in PB (**a**), spleen (**b**), and BM (**c**) during leukemia development. These results were generated by the optimized parameters. Exp, Experimental data; Sim, Simulation results; *d*_*1,2*_, Model fitness degree (d_1_- leukemic, d_2_- normal).; *s*_*1,2*_, Sample standard deviation (*s*_*1*_ - leukemic, *s*_*2*_ - normal). (**d**–**e**). Computational dynamics of HSCs (D) and HPCs (E) with the optimized parameters

The counts of normal hematopoietic (CD45.1^+^) and leukemic (GFP^+^) cells in peripheral blood (PB), spleen and BM were monitored and documented from the experimental mouse model at indicated time points (Additional file 1: Figures S1A-S1C) [11]. As shown, GFP^+^ leukemic cells grew dramatically during leukemia development in all these tissues. The number of CD45.1^+^ normal cells in PB had obvious increase after day 7, resulting in the elevation of white blood cell (WBC) counts at day 14 (Additional file 1: Figure S1A). Meanwhile, the number of CD45.1^+^ cells in spleen slightly increased before day 14, but reduced by day 21 (Additional file 1: Figure S1B). In contrast, the leukemic BM underwent a modest decrease in cellularity, and the number of CD45.1^+^ cells was gradually reduced, reaching a minimum at day 21 (Additional file 1: Figure S1C). Uitlizing the experimental data as reference, we established a computational model that was able to mimic the tri-tissue hematopoietic dynamics under leukemia. The model variables corresponded to the normal and leukemic cell counts in the different tissues; and dynamics of the variables depended on the actions of cellular mechanisms (eg cell proliferation, mobility, death, etc.), which were encoded by kinetic rate formulas and leukemic influences were considered. Mathematical forms of the kinetic rates were adopted from previous studies [12–14], and parameters were fitted based on the reference data of experimental cell kinetics. Effective optimization methods were adopted in the parameter fitting (Methods), and the high fitness illustrated that the model parameters (as well as the formulas) were reasonably estimated (Fig. 2a–c). Therefore, the model was capable of simulating the cell counts in any/all tissues at arbitrary time points (ie hematopoietic dynamics) for the leukemic body.

#### Sub-model for the hematopoietic stem/progenitor cell (HSC/HPC) dynamics in the leukemic environment

Because of the necessity for investigating the hematopoietic primitive cells (ie HSC/HPC), we also extended the computaitonal model to enclose the HSC/HPC in BM. Although these cells might also reside and grow in other tissues, BM was undoubtedly the major location for hematopoietic primitive cells; and the BM HSC/HPC actually accounted for the large majority of hematopoietic functions [15]. Again, we utilized the cell counts of HSC (Lin^−^c-Kit^+^Sca1^+^, LKS^+^) and HPC (Lin^−^c-Kit^+^Sca1^−^, LKS^−^), which amounted to a portion in the BM CD45.1^+^ population (Additional file 1: Figures S1D–S1E), as the reference data; and the extended model for HSC/HPC dynamics was established in the similar way as mentioned earlier (Methods). We estimated the model parameters and a high fitness was achieved, implying that the parameter optimization was successful (Fig. 2d–e). As shown in the cell kinetics, a suppression of hematopoiesis was indicated during leukemia development as both HSC and HPC in BM were reduced dramatically.

### Model validation

#### Reproduction for control

A preliminary test for the model validity would be checking whether the model could correctly reproduce the normal (ie disease-free) kinetics when the parts representing leukemic influences in the model were directly removed (ie corresponding parameters purged to zeros without changing anything else), which was supposed to be satisfied by a valid model in the first place. By simply removing the leukemic-effect terms from the model while keeping everything else unchanged, we could see that the normal kinetics, ie, hematopoietic cells as well as HSCs/HPCs were maintained at nearly steady levels [11], were reproduced (Additional file 2: Figure S2). Thus the model not only accurately represented the leukemia-conditioned hematopoietic dynamics; it could also faithfully reflect the situation under the normal condition. Therefore, the risk of overfitting for the leukemia-conditioned dynamics had been lessened; in other words, the model was likely to have captured the generic characteristics of hematopoiesis, rather than artificially-fitted phenomena.

#### Identifying the major cause of HSC loss in leukemia

**Fig. 3:**
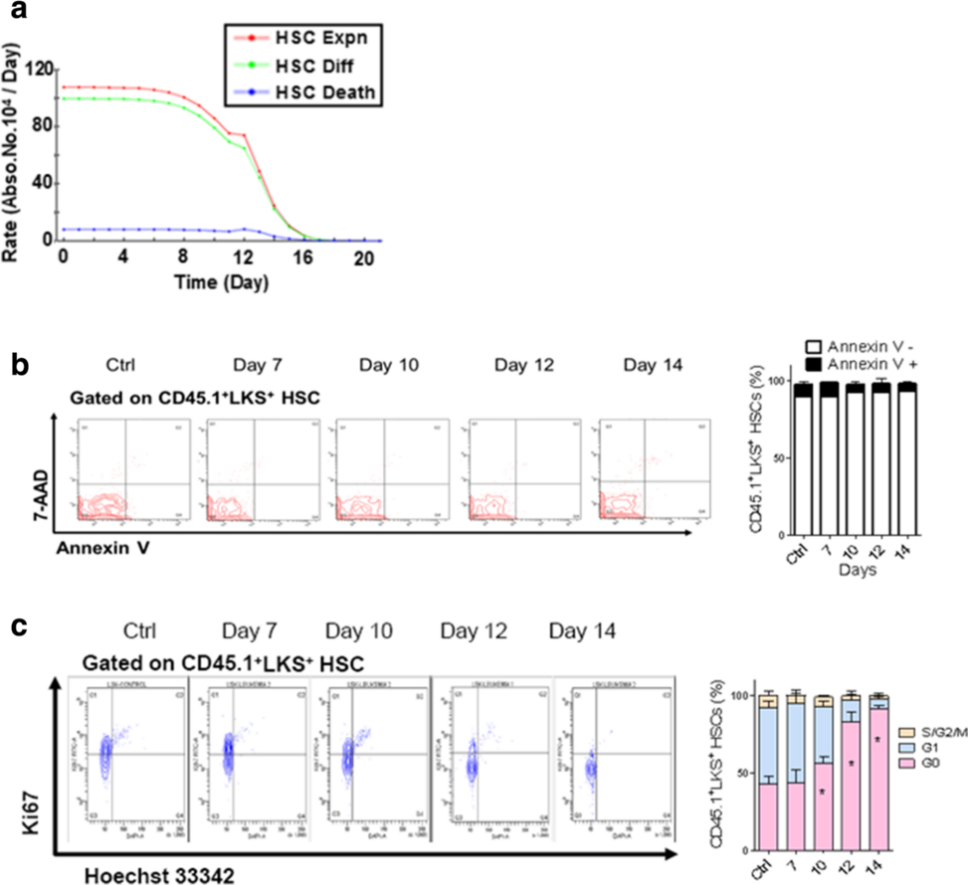
Major factor of HSC loss validated by cell cycle and apoptosis analyses of HSCs in leukemic BM. **a** Computational decomposition of the HSC dynamics on the relevant processes. Cell expansion (Expn, red), differentiation (Diff, green) and death (Death, blue). **b** Flow plots (left panel) and histogram (right panel) show the apoptosis frequency of HSCs (LKS^+^) in leukemic BM [11]. **c** Flow plots (left panel) and histograms (right panel) show the cell cycle status of HSCs in leukemic BM. Data are represented as the mean ± SEM (*n* = 12; 3 independent experiments); ^*^ *p* < 0.05 [11]

The BM HSC (LKS^+^) level was almost constant under normal conditions but decreased in leukemia (Fig. 2d and Additional file 1: Figure S1D). With the advent of the computational model, we could systematically investigate the relevant factor(s) for the altered HSC kinetics computationally. Hence, we intended to determine which cellular mechanism (eg, expansion, differentiation or cell death) was primarily responsible for the HSC loss during leukemia development. To this end, parametric analysis (Methods) was implemented to compute all action rates of HSCs including cell expansion (*f*_expn_), differentiation (*f*_diff_) and death (*f*_death_). The analysis revealed that *f*_expn_ and *f*_diff_ were large in both value and variation amplitude, whereas the value and variation amplitude of *f*_death_ were small (Fig. 3a). Therefore, the change in HSC level was mainly due to HSC expansion or differentiation but not cell death. As *f*_expn_ and *f*_diff_ are achieved or associated with “proliferation” via the cell cycle and cell death is mainly induced by apoptosis, we concluded that the major factor for HSC loss was an alteration in cell cycle rather than apoptosis. This analysis could be better visualized in a 3-dimensional form, displaying *f*_expn_, *f*_diff_, and *f*_death_ on the three dimensions (Additional file 3: Figure S3A). Noteworthy, the computed rate for HSC proliferation decreased with time, with rapid decline occurring around day 10; this suggested that HSCs became much more quiescent as leukemia cells evolved as a dominant population (Fig. 3a).

The computational prediction was consistent with the experimental results of flow cytometry analyses [11]. In fact, the Annexin V and 7-aminoactinomycin D (7-AAD) staining, which was for the assessment of apoptosis, revealed that the ratio of Annexin V^+^ cells in BM HSCs (LKS^+^) was nearly unchanged and actually accounted for a small percentage during leukemia development (Fig. 3b). In contrast, the intracellular Ki67 and Hoechst 33342 staining, which was designed for analyzing the cell cycle of HSCs in leukemic BM, showed that the cell cycle status of HSCs changed significantly. Notably, the proportion of HSCs in the G0 phase (Ki67^−^, 2 N DNA content) progressively increased during leukemia development, with a large climb occurring around day 10 (Fig. 3c), which coincided with the computational result of proliferation rate decline (Fig. 3a). Interestingly, HSCs in the late stages (after day 14) of leukemia remained in a nearly non-proliferative state (>92 % LKS^+^ cells were in G0 in comparison with 40 % from the control mice, Fig. 3c), as further confirmed by a BrdU incorporation assay (Additional file 3: Figure S3B). Taken together, our computational results on the deepened HSC quiescence were validated by independent cellular assays. Therefore, we concluded that the suppression of HSCs in leukemic BM was mainly due to the functional alteration of cell cycle, not apoptosis.

#### Simulating the hematopoietic differentiation blockade

**Fig. 4:**
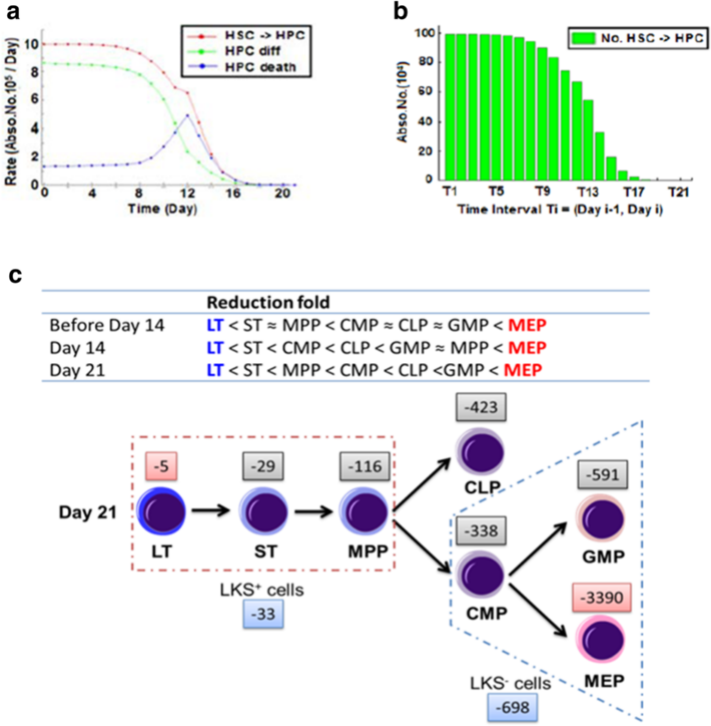
Hematopoietic differentiation blockade validated by reduction folds in different stem and progenitor subsets in the hematopoietic cascade. **a** Computational decomposition of the HPC dynamics on relevant processes. HPC generation (ie HSC differentiation into HPC – HSC- > HPC, red), HPC differentiation (Diff, green) and death (Death, blue). **b** Absolute numbers of HSCs differentiating towards HPCs in consecutive time intervals simulated by the computational model. *T*_*i*_ is [day *i*-1, day *i*] for *i* = 1, 2, …, 21. **c** Reduction folds in different HSC/HPC subsets (upper panel) and an experimental schema (lower panel) showing the differentiation block from HSCs to HPCs. Numbers −5, −29, etc. indicate the fold decrease [11]

Given the poor production of blood cells in the late stages of leukemia development, we further extended our parametric analysis to the HPCs. We computed the action rates of HPCs, namely, HPC generation (ie HSC differentiation into HPC, *f*_HSC->HPC_), HPC differentiation (*f*_diff_) and death (*f*_death_). The results showed that between the two factors having both large values and variations, *f*_HSC->HPC_ overrode *f*_diff_ at all times and *f*_diff_ decayed much faster than *f*_HSC->HPC_, which occurred between days 10 and 12 (Fig. 4a). After day 10, the HPC kinetics was largely dictated by the changes in *f*_HSC->HPC_, suggesting that *f*_HSC->HPC_ was the major cause of HPC loss or exhaustion. Therefore, a blockade of hematopoietic differentiation was implicated. Meanwhile, *f*_death_ rose moderately around day 12, implying that there might be other cell death-related events contributing to the loss of HPC.

To further demonstrate and visualize the blockade of differentiation, we computed the absolute numbers of HSCs differentiating into HPCs in consecutive time intervals (Methods). Notably, there were indeed fewer and fewer HSCs differentiating toward HPCs during leukemia progression, and the number was almost vanished by day 21 (Fig. 4b). This clearly revealed the shutdown of HSC differentiation, which caused that HPCs could not be replenished in leukemic BM. On the other hand, BM HPCs need to produce mature blood cells; therefore, BM HPCs suffered a much sharper decline than BM HSCs and ultimately became exhausted in leukemic BM (Additional file 1: Figures S1D–S1E).

If the above prediction were true, we should then expect that the reduction of HSCs/HPCs was correlated with the hierarchy of differentiation in the hematopoietic cascade. To this end, data of the whole spectrum of different subsets of HSC/HPC were adopted [11]. As shown in the data, three subsets of LKS^+^ HSCs, namely, LT-HSCs (LKS^+^CD34^−^ or LKS^+^CD150^+^CD48^−^), ST-HSCs (LKS^+^CD34^+^Flk2^−^) and MPPs (LKS^+^CD34^+^Flk2^+^), all suffered progressive decreases in absolute numbers during leukemia development (Additional file 4: Figure S4A). Meanwhile, the absolute numbers of all four subsets of LKS^−^ HPCs in leukemic BM, namely CMPs (LKS^−^CD34^+^CD16/32^−^), GMPs (LKS^−^CD34^+^CD16/32^+^), MEPs (LKS^−^CD34^−^CD16/32^−^), and CLPs (LK^low^S^low^IL7Rα^+^), were greatly reduced during leukemia development (Additional file 4: Figures S4B-S4C). Obviously, among all of the subsets of primitive cells, LT-HSCs were the least reduced while the MEPs were the most reduced at different stages of leukemia (Fig. 4c, Additional file 4: Figure S4D). In addition, it was also confirmed that LT-HSC became much more quiescent in leukemia (Additional file 4: Figure S4E–S4F). Therefore, the computational prediction here was confirmed; and altogether, these results demonstrated that leukemic BM resulted in a blockade of hematopoiesis in a differentiation-dependent manner.

### Further computational analyses

#### Systematicity rather than individuality of modeling

**Fig. 5:**
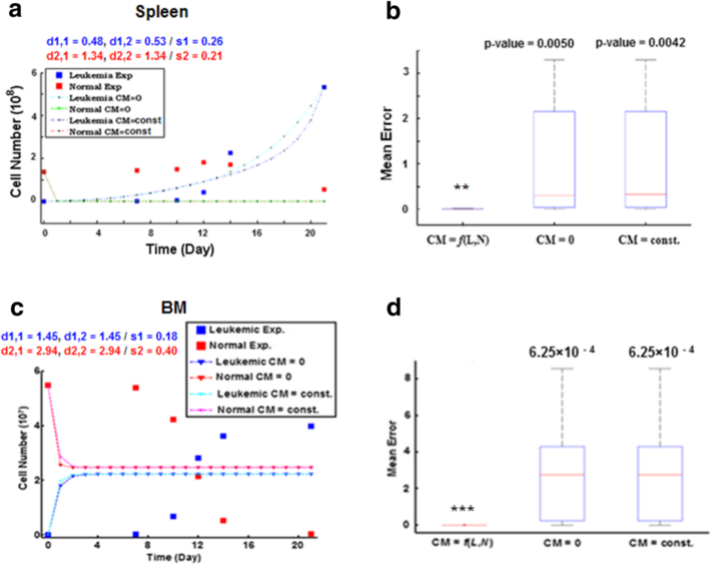
Comparisons of model fitness between systematic and individual modelings. **a** Inferior fitness of the plain models for the spleen cell kinetics, which ignore the inter-connections between tissues (cell mobility, *CM* = *0*) or overlooking them as a constant (*CM* = *const*) unrelated with PB kinetics. *d*_*1,1*_, *d*_*1,2*_ - fitness degrees for leukemic kinetics when *CM = 0* and *const,* respectively; *d*_*2,1*_, *d*_*2,2*_ - fitness for normal cell kinetics when *CM = 0* and *const. s*_*1,2*_, Sample standard deviation (*s*_*1*_ - leukemic, *s*_*2*_ - normal). **b** Box plots illustrate a significantly higher fitness of the model including the CM as a function of PB kinetics (ie *CM* = *f*(*L*,*N*), *L* - PB leukemic cells, *N* - PB normal cells) for the spleen cell kinetics (one-tail *t*-test, ^**^ *p* < 0.005). **c** Inferior fitness of the plain models ignoring or overlooking cell mobility for the BM cell kinetics. **d** Significantly higher fitness of the model including CM as a function of PB kinetics for the BM cell kinetics (one-tail *t*-test, ^***^ *p* < 0.001)

In silico experiments revealed that cell mobility (ie inter-connections between tissues) was important for modeling the dynamics of both leukemic and normal cells. In fact, modeling revealed an improvement of fitness for the observed kinetics in both spleen and BM when cell mobility was included in the model (ie cell mobility was formulated as a function of the PB circulation states); whereas the plain models disregarding cell mobility, ie ignoring it or overlooking its effect as a constant unrelated with PB kinetics, exhibited significantly higher errors (Figs. 5a–d). Hence, the strategy for modeling individual tissues stipulated in most of previous studies was unable or insufficient for reconstructing the complex multi-tissues dynamics in the leukemic body. In other words, the necessity of studying the diseased hosts by systematic approaches was reinforced.

#### Differential cell kinetics in three tissues

**Fig. 6:**
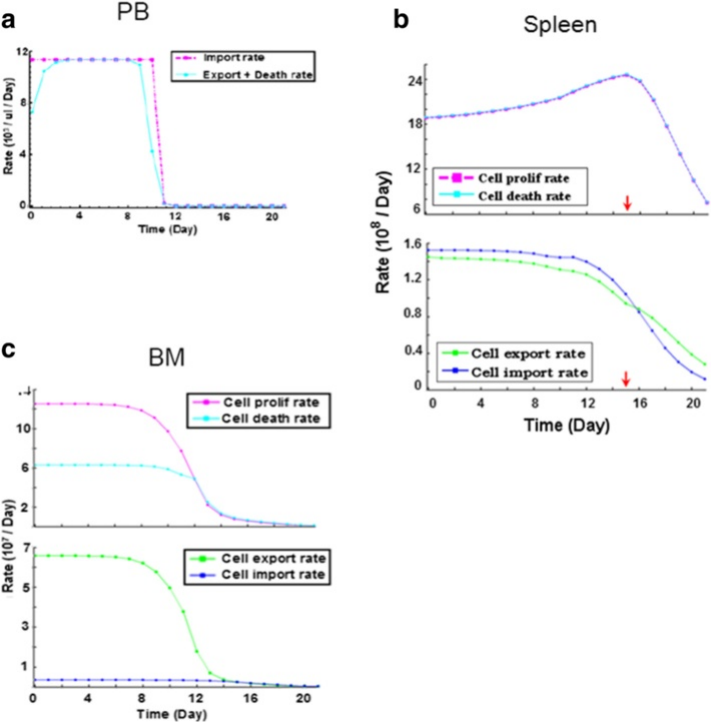
Parametric analyses for differential cell kinetics in PB, spleen and BM. **a** Action rates of normal cells at the tissue-level (ie total CD45.1^+^ cells) in PB during leukemia development. As primitive blood cells rarely occur in PB circulation, the PB cells are regarded as having no proliferation. **b** Actions rates of normal cells in spleen during leukemia development. Upper panel: proliferation and death rates for total CD45.1^+^ cells in spleen; lower panel: cell export and import rates; red arrow: time point when the number of spleen CD45.1^+^ cells reaches maximal. **c** Actions rates of normal cells in BM during leukemia development. Upper panel: proliferation and death rates for total CD45.1^+^ cells in BM; lower panel: cell export and import rates

By parametric analysis of the model, the rate of each cell action was simulated; and we computationally found that the differences of cell kinetics in the respective tissues were underlain by the asymmetric leukemic influences on the cell actions in different tissues. In PB, the cell import rate (*r*_Import, PB_) was larger than (or equal to) the sum of export rate (*r*_Export,PB_) and death rate (*r*_Death,PB_) for almost all the time (Fig. 6a); thus having explained the increase in the PB CD45.1^+^ cell number (Fig. 2a and Additional file 1: Figure S1A). The kinetics of hematopoietic cells in spleen exhibited a short increase at early stage of leukemia (Fig. 2b and Additional file 1: Figure S1B). In fact, the cell proliferation rate (*r*_Prolif,SP_) and death rate (*r*_Death,SP_) were nearly equal; and the cell import rate (from PB; *r*_Import,PB->SP_) was slightly larger than the cell export rate (*r*_Export,SP->PB_) in an early period of leukemia (Fig. 6b); in addition, *r*_Import,PB->SP_ decreased slower than *r*_Export,SP->PB_. Thus the early increase of spleen cell counts was resulted in. Here we could see that the pivotal underlying factor is *r*_Import,PB->SP_, since although *r*_Prolif,SP_ is increasing, *r*_Death,SP_ almost has the same trend. Moreover, the facts that “*r*_Prolif,SP_ increased” and “*r*_Import,PB->SP_ > *r*_Export,SP->PB_” supported a hypothesis that spleen might undergo a compensatory mechanism in response to leukemia. On one hand, it increased cell proliferation; on the other hand, the cell importation became larger as hematopoietic cells migrated from elsewhere via PB circulation (due to the malignancy stress, ie destruction of microenvironments) might be restored in spleen at the onset of leukemia.

In contrast, the BM cell proliferation rate (*r*_Prolif,BM_) decreased monotonously and the cell export rate (*r*_Export,BM->PB_) was greater than the import rate (*r*_Import,PB->BM_) during leukemia development (Fig. 6c). The decreased proliferation could be explained by the suppression of hematopoiesis in leukemic BM (HSC/HPC losses, Fig. 2d–e and Additional file 1: Figure S1D-S1E); and the large *r*_Export,BM->PB_ indicated that cells migrated out of the BM as the environment became worse during leukemia progression. Notably, at late stages of leukemia, *r*_Export,BM->PB_ dropped because BM cells were depleted as influenced by leukemia, thus fewer normal cells could escape the malignant environment.

Altogether, the different cell kinetics in the respective tissues were due to the asymmetric influences that leukemia exerted on the cell actions; and as shown during the parametric analyses, the key factors were the cell export/import rates. Therefore, because the rates of cell export/import were pivotal in shaping the different cell kinetics and cell mobility was of great importance to modeling (stated earlier), processes of cell trafficking between different tissues in the diseased host might also be valuable for studying the disease, in addition to the investigations of leukemic or hematopoietic cells themselves.

### Possible G0 Re-entry of HSCs for increased quiescence-implication from model selection

**Fig. 7:**
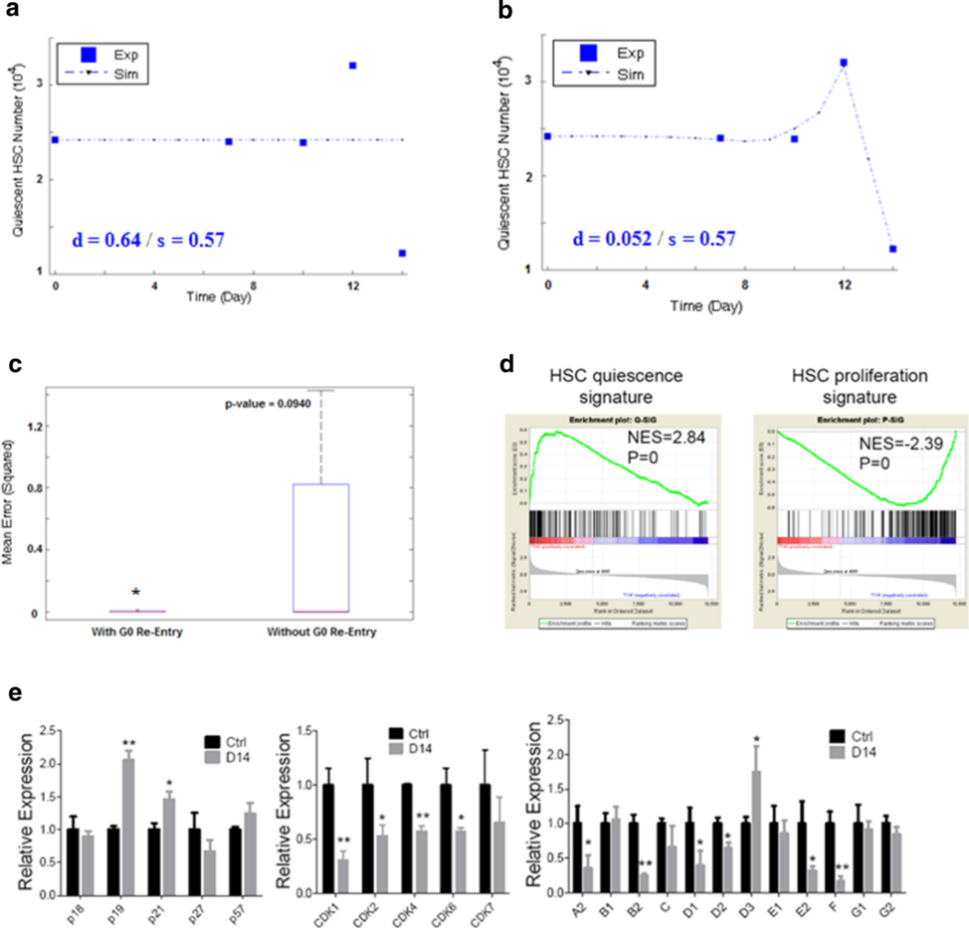
Model-selection implication for G0 re-entry of HSCs in leukemic BM. **a** Failure of the plain model, which is not encoding G0 re-entry, for representing the kinetics of quiescent HSCs. **b** Success of the model including the G0 re-entry for representing the kinetics of quiescent HSCs. Here we show one specific example in which the G0 re-entry is formulated by the most common first-order kinetics, ie linear form. See also Additional file 5: Figure S5 for supplemental results where the G0 re-entry adopts other forms. **c** Box plot illustrating that the fitness of the model with G0 re-entry is higher than the plain model (one-tail *t*-test, ^*^ *p* < 0.1). See also Additional file 5: Figure S5 for supplemental results in which models with (other forms of) G0 re-entries uniformly have higher fitness degrees than the plain model. **d** GSEA for BM HSCs from day 14 leukemia and control mice. The up-regulation or down-regulation of quiescence-associated gene expressions (left panel) and proliferation-associated gene expressions (right panel) are shown. The normalized enrichment scores (NES) and *p*-values are indicated in each plot [11]. **e** The histograms show cell cycle-related gene expressions in BM HSCs at leukemia day 14 compared to control. CKIs (left panel), CDKs (middle panel) and cyclins (right panel) were shown. Data are represented as the mean ± SEM (*n* = 3, 3 independent experiments), ^*^ *p* < 0.05, ^**^ *p* < 0.01 [11]

The apparent increase in the percentage of quiescent HSCs in leukemic BM might involve two possible scenarios: (i) cycling HSCs were selectively exhausted or (ii) more cycling HSCs became quiescent (G0 re-entry). Since it was difficult to tackle the issue directly by in vivo experiments, we once again utilized computational modeling to evaluate which scenario was more likely (Methods). We found that the model fitness for the observed HSC cell cycle kinetics was improved when G0 re-entry was included (Fig. 7a, Additional file 5: Figures S5A–S5D), whereas the counterpart model without G0 re-entry exhibited larger errors and failed to represent the kinetics of quiescent HSCs (Fig. 7b–c). Therefore, the results showed that scenario (ii) was better correlated with the experimental data, meaning that a mechanism causing cycling HSCs to become quiescent was more likely in response to the leukemic stress.

Although direct experimental validation for the existence of G0 re-entry was difficult, gene expression profiling and gene set enrichment analysis (GSEA) revealed that expressions of quiescence-associated genes were significantly enriched in the HSCs from leukemic hosts, while expressions of proliferation-associated genes were markedly depleted (Fig. 7d). Futhermore, qRT-PCR confirmed that in HSCs from leukemic hosts, p19^INK4d^ and p21^Cip1^ were upregulated, and virtually most CDKs and cyclins were suppressed (Fig. 7e) [11]. Therefore, we could conclude that normal HSCs in leukemic BM were functionally suppressed during leukemogenesis as they were forced to be in a more quiescent status.

Moreover, the recent work of Miraki-Moud et al. had also established an equal conclusion that AML did not demolish normal HSCs but induced arrest or immobilization of cell cycle by impeding their differentiation [16]. Thus it was actually equivalent to a further corroboration for our main conclusions (ie major cause of HSC loss, hematopoietic blockade, and increased quiescence of HSC under leukemic stress) in overall.

## Discussion

Researches of mathematical modeling for leukemia have been undertaken for years [13, 14, 17, 18]; while the previous studies are informative as they have shown the feasibility of quantitatively and dynamically modeling the cell kinetics in leukemia, there are also important issues overlooked that preclude better investigation for the system of a leukemic body. For instance, the large majority of previous studies focused on only leukemic cells or single tissues, whereas few (or none) of them was able to simultaneously model the hematopoiesis fulfilled by both leukemic and normal blood cells in multiple tissues of a diseased host. With the aid of an un-manipulated animal model resembling the physiological condition of leukemogenesis (ie intact normal blood cells without overt damages can be directly measured during leukemia development), a comprehensive computational model that embodies the multi-tissue dynamics of both leukemic and normal cells in the leukemic environment, can be established via a systematic modeling approach. In this study, we proceeded the modeling in a hierachically-systematic manner; we first derived a model on the tissue-level (ie total hematopoietic cells with respect to the multiple tissues), and then we used the derived model as a basis for building the sub-model of HSCs/HPCs, with inter-connections of PB with spleen and BM fully considered. Finally, the HSC sub-model had been splitted into a even more microscopic scale, in which quiescent and active HSCs were distinguished so that HSC quiescence could be explored.

Specifically, the key step in the modeling is parameter optimization. A model will be operational only after all its parameters are reasonably estimated, which means that the results produced by the model with the estimates fit the reference data. But rigorously, even a good model fitness is obtained (with a certain reference data), the model validity has not come into being, since the validations must be achieved by additional (independent) experimental data. Since HSCs/HPCs are of the primary interests in hematology and experiments with respect to them are intensive, we therefore run parametric analyses on them to assess the influential factors for their suppression (or loss) during leukemia development. Actually, parametric analysis is equivalent to decomposing the cell kinetics, ie net-effects resulting from actions of various factors, onto each particular factor. Thus the major one(s) for the altered hematopoietic kinetics under leukemia can be found. After performing the analyses on HSCs/HPCs, ie simulating the action rates associated with them, the major cause for HSC loss was identified (Fig. 3); meanwhile, a hematopoietic differentiation blockade having explained the exhaustion of HPCs was observed (Fig. 4). Both predictions have been validated by additional experimental data [11]; and moreover, another independent work has also supported our main conclusions [16], thus our computational model can be credited for validity.

During the modeling, we have had several interesting findings from the computational viewpoint. First, by working with the recent data of multiple tissues in leukemic hosts, we found that the principle of individuality stipulated in majority of previous modelings no longer worked for a multi-tissue system. To reconstruct the correct cell kinetics in different tissues simultaneously, the PB circulation must be considered and explicitly modeled. For example, if we neglected it or simply assumed it to be constant term unrelated with the PB kinetics, large errors were resulted in. In contrast, if we considered this term to be a function of the kinetics of the PB leukemic/normal cells, even with the simplest form (as described in Methods), the modeling errors were significantly reduced immediately, for the cell kinetics in both spleen and BM (Fig. 5). Second, parametric analyses for the cell actions on the tissue-level (ie simulations for the actions of total hematopoietic cells in different tissues) indicated the reasons underlying the differential kinetics in PB, spleen, and BM. The computational findings that the activation of spleen hematopoiesis and increase of spleen cell influx (at the onset of leukemia), PB cell counts (WBC) elevation, and the continuous depression of BM hematopoiesis, suggested the possible response of the host to leukemia in general (Fig. 6).

Last but not least, we derived a hypothesis via model selection, which implied that the increased quiescence of HSCs might be because of re-entry to the G0 phase under leukemic stress. Nonetheless, it was not unaware of that the p-value for the model selection was not quite ideal (Fig. 7a–c); this was because of the scarcity of data. As shown, there were only five data points to be fitted, and the first three were just nearly equal since the normal HSCs still maintained steady levels at early stage of leukemia (Fig. 7a–b). Therefore, even the false model could automatically have a fitness of three out of the five reference data; thus the visual discrimination between the superior and inferior models were not so impressive. But obviously, the inferior model could not describe the kinetic profile of quiescent HSCs in total; thus we could speculate that if there were more data points existing, the contrast between the two models would be stronger. Moreover, on the other hand, the modeling using only five data points generated the significance level of *p*-value < 0.1^*^; we could well speculate that even if there were only one more data spot between day10 and day 12 and it had rising trend as that of day 12, the *p*-value would be markedly lowered. Hence, although we could not assert the existence of G0 re-entry based on only the modeling results herein, these results could still propose a testing method-we could re-perform the cytometry experiment to measure the HSC counts between day10 and day 12, and if we found that the number of quiescent HSCs rose above, the hypothesis of G0 re-entry would be strengthened. Given the facts that the quiescent HSCs are relatively insensible to environmental influences and also remained highly functional when transplanted into secondary non-leukemic hosts [4, 11, 16], we believe that acquired quiescence of normal HSCs represents a cellular protective mechanism in response to leukemia. Naturally, more detailed studies for the quiescence of HSCs must be subjected to future experiments.

On the other hand, our computational model might be of valuable utilities. For example, the model as well as its simulations could be stored in computers (or databases); thus if an animal model of the same diseased condition is needed, we will not have to build it again or take the real measurements, we can just refer to the digitalized model to retrieve the data for a preliminary assessment or investigation. Therefore, the experimental costs can be saved. Moreover, such a digitalized model can also serve as a virtual platform for simulating the efficacies of drugs or interventions, facilitating the computer-aided researches for disease therapies.

## Conclusion

In this study, we have digitalized an experimental non-irradiated AML mouse model that is recently established. The computational model has been validated with additional experimental data from multiple aspects; and given its validity, it may be of potential utilities for future biology or biomedical researches. Meanwhile, the modeling method can also be applied otherwhere. Moreover, our computational conclusions, altogether with the experimental supports, provide evidences for the increased quiescence of BM HSCs and hematopoietic suppression/blockade under leukemia, which is an attractive perspective in current hematology. These findings may also provide insights for more extensive studies, eg therapeutics of stem cells, or interventions for leukemia.

## Methods

### Experimental model and data

Data of cell kinetics were adopted from the recently-established non-irradiated AML mouse model [11]. In this experiment, counts of normal hematopoietic (CD45.1^+^) and leukemic (GFP^+^) cells in PB, spleen and BM were measured at day 0, 7, 10, 12, 14 and 21. The sub-populations of HSCs (CD45.1^+^LKS^+^) and HPCs (CD45.1^+^LKS^−^) in BM were also specifically quantified. Specifically, tissue samples were taken from the (non-irradiated) AML mice at the indicated time points, and flow cytometry was applied to sort the populations of different cells. Normal and leukemic cells were sorted by gating on CD45.1+ and GFP+ respectively. HSCs and HPCs were further sorted by gating on the surface markers Lin- Sca-1+ c-Kit + (LKS+) and Lin- Sca-1- c-Kit + (LKS-) in the CD45.1+ population, according to a protocol for broad classification [19, 20]. Frequencies of different cells in the respective populations, as well as the absolute cell numbers, were recorded thereby. Besides the data of cell kinetics, additional data of cell cycle and apoptosis analyses of BM HSC, flow cytometry analyses of HSC/HPC subsets in leukemic BM, and gene expression analyses, were also provided [11]. These data were utilized as independent evidences for model validation.

### Mathematical modeling

Our model was an analog of a three-compartment system containing particles undergoing various processes of proliferation, decay, transportation, and etc. Specifically, spleen and BM were two compartments and PB was a third one that directly connected them; due to PB circulation, the inter-connection between BM and spleen was via PB. Complying with cell biology, there were at most four processes influencing cell dynamics within a tissue, namely, cell proliferation (*Prolif*), death (*Death*), and migrations out of/into the tissue (*Export*/*Import*) due to cell mobility. Meanwhile, noise was reasonably omitted as the numbers of cell are massive (above 10^3^ ~ 10^4^). Therefore, the dynamics of cell number (*X*) in an arbitrary population (ie, *dX*/*dt*) could generically be a mathematical form (Eq. 1):

1 $$ \frac{dX}{dt}={f}_{Prolif}-{f}_{Death}-{f}_{Export}+{f}_{Import} $$

where the right-side functions were kinetic rates of the respective cellular actions (ie acting factors of cell dynamics).

Cells in different populations or tissues might undergo different processes and their actions might follow different kinetic laws; moreover, the actions of hematopoietic cells were also subjected to leukemic influences. For detailed descriptions, refer to Additional file 6.

### Numerical optimization for model formulas/parameters

As argued by Almquist et al., although the simultaneous optimization for both unknown parameters and uncertain formula structures would be extremely difficult, by finite enumeration of model structures, the complex model optimization was reduced to an ordinary issue of parameter estimation [21]. Therefore, we assumed that the rates of cellular actions might follow any of the possible kinetic forms, eg the zeroth-order kinetics, first-order kinetics, exponential kinetics, Michaelis-Menten and Hill kinetics, etc., which had been studied and adopted in previous researches to approximate complex biochemical/biological processes [22–24]. Additionally, the influences of leukemic cells could adopt the Hill form, as proposed by previous studies [13, 24]. We successively substituted the specific kinetic forms into each part of Eq. 1, and thus a pool of dynamical equations with different formulas was generated. Then, we selected the best numerical fit(s) in this pool with the aid of appropriate numerical algorithms of optimization.

The reference data utilized in the parameter optimization were the cell kinetics measured from the experimental leukemia model [11]. We used Genetic Algorithm (GA) as the main approach because it is a heuristic method capable of alleviating computational complexity [25]. In addition, other algorithms (such as the trust-region method) were also applied as complements, since hybrid usage of them with GA might enhance the computation efficiency [25]. The criterion was that the mean deviation of the computed values with respect to the optimized parameters (defined as the root-mean-square deviation, *d*) was no larger than the experimental standard deviation (*s*), ie, *d* ≤ *s*.

### Parametric analysis

After completion of the model, we could simulate the continuous system dynamics with equation-solving approaches (eg the Gear method). We then utilized the simulations to calculate the cell action rates (right side of Eq. 1), which amounted to projecting the cell dynamics (left side of Eq. 1) on different acting factors corresponding to cellular mechanisms or bio-processes. In fact, for a dynamic process depicted by Eq. 1, the projection of the variation rates of cell numbers (at arbitrary time *t*) on a single acting factor directly indicated how much this term caused the cell number to change (at time *t*). In this manner, contributions of different factors could be assessed and the major one(s) could be found. Generically, given the simulation results of hematopoietic and leukemic cell dynamics {*X*(*t*_*j*_) : *j* = 0,1, …} and {*L*(*t*_*j*_) : *j* = 0,1, …}, the specific rate (at arbitrary *t*_*j*_) of any cellular action for hematopoietic cells could be calculated as:

2 $$ {f}_i\left(X\left({t}_j\right),K\left(L\left({t}_j\right)\right)\right),i\in \left\{ Prolif, Death, Import, Export, Expn, Diff\right\} $$

where *f*_*i*_ were the kinetic forms and *K* was a function encoding leukemic influences as mentioned earlier.

### Model selection

Using the model framework as the working platform, we could in silico evaluate different (usually opposite) scenarios; eg, the necessity of cell mobility, and the possibility of G0 re-entry. Generally, different equations could be formulated according to whether a hypothesis or its null counterpart was adopted. Thus two models distinct from each other were formed, eg with or without cell mobility/G0 re-entry. Statistical tests were then performed to examine which model possessed superior fitness for the (given) reference data; and the selected model was assumed to be more likely to have produced the experimental data. In the present work, the one-tail *t*-test was used to check whether the mean error resulted by one model was significantly lower (or higher) than that of the other in the fitting of reference data. For more details, refer to Additional file 6.

## Additional files

Additional file 1: Figure S1. Raw data of cell kinetics from experiment. (A–C). Absolute numbers of CD45.1^+^ normal hematopoietic cells and GFP^+^ leukemia cells in PB (A), spleen (B) and BM (C) during leukemia development (*n* = 5-7). Ctrl, Control mice [11]. (D–E). Absolute numbers of CD45.1^+^LKS^+^ (D) and CD45.1^+^LKS^−^ (E) cells in leukemic BM (*n* = 4-5) [11]. (PNG 71 kb) Additional file 2: Figure S2. Reproduction of the normal control by the model. (A–C). Computational and experimental cell kinetics of PB (A), spleen (B) and BM (C) under the normal condition. The computation results are yielded by directly eliminating the parameters for leukemic effects in the mathematical model. Experimental data are taken from Ref [11]. (D). Computational and experimental kinetics of BM HSCs/HPCs under the normal condition. (E). Computational and experimental kinetics of quiescent and active HSCs in BM under the normal condition. (PNG 79 kb) Additional file 3: Figure S3. Supplemental data for illustrating the major factor of HSC loss. (A). The 3D visualization of projections of the HSC dynamics. Expn, Diff and Death are the orthogonal axes; and the rates projected on them are bars with lengths proportional to the values. Prolif is symbolized as the vectorized composition of Diff and Expn. Temporal profiles at day 0, 7, 10, 12, 14 and 21 are given. (B). Flow plots (left panel) and histogram (right panel) show the BrdU incorporation of HSCs (LKS^+^ cells) in leukemic BM. Data are represented as the mean ± SEM (*n* = 8, 2 independent experiments). ^***^ *p* < 0.001 [11]. (PNG 165 kb) Additional file 4: Figure S4. Supplemental data for illustrating the differentiation blockade in the hematopoietic cascade. (A). Absolute numbers of LT-HSCs, ST-HSCs and MPPs in leukemic BM [11]. (B–C). Absolute numbers of CMPs, GMPs, MEPs (B) and CLPs (C) in leukemic BM. Data are represented as the mean ± SEM (*n* = 12, 3 independent experiments). ^*^ *p* < 0.05, ^**^ *p* < 0.01, ^***^ *p* < 0.001. + or -, increase or decrease [11]. (D). A pattern showing linear correlation between the reduction and differentiation hierarchy in the hematopoietic cascade. The reduction of LT-HSC was normalized to −1, and the bars indicate normalized reduction level [11]. (E). Flow plots (left panel) and histograms (right panel) show the cell cycle status of LT-HSCs in leukemic BM. Data are represented as the mean ± SEM (*n* = 12, 3 independent experiments). ^*^ *p* < 0.05 [11]. (F). Flow plots (left panel) and histogram (right panel) show the BrdU incorporation of LT-HSCs in leukemic BM. Data are represented as the mean ± SEM (*n* = 8, 2 independent experiments). ^***^ *p* < 0.001 [11]. (PNG 135 kb) Additional file 5: Figure S5. Supplemental data for illustrating the increased quiescence of HSCs. (A-B). Fitness of the models including the G0 re-entry, which adopts two other forms: the Hill form (A) and exponential form (B). Reference data for the kinetics of quiescent HSCs were taken from the cell cycle analyses (Fig. 3c) [11], in which percentage numbers (G0) were transformed into absolute numbers by multiplying the total cell count of HSC population (CD45.1^+^LKS^+^, Additional file 1: Figure S1D). (C–D). Box plots show that the mean errors of the models with G0 re-entry are uniformly lower than the plain model (one-tail *t*-test, ^*^ *p* < 0.1). (PNG 54 kb) Additional file 6: Descriptions in-detail for the computational model. Descriptions of the modeling process and computational procedures are enclosed; concrete mathematical formulas and (optimized) parameter values are also provided. (DOCX 152 kb)
