# Supplementary figures and images for: Digitalization of a non-irradiated acute myeloid leukemia model
Source: BMC Syst Biol. 2016 Aug 26;10(Suppl 3):64. doi: 10.1186/s12918-016-0308-x (PMC5009825; doi:10.1186/s12918-016-0308-x)

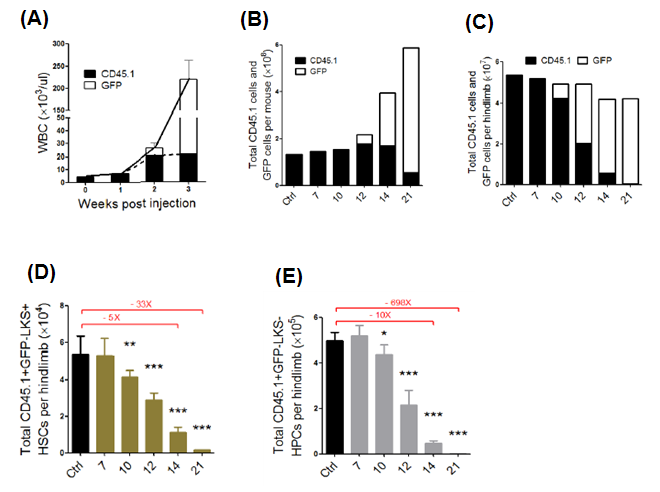

Supplement: Additional file 1: Figure S1. — Raw data of cell kinetics from experiment. (A–C). Absolute numbers of CD45.1+ normal hematopoietic cells and GFP+ leukemia cells in PB (A), spleen (B) and BM (C) during leukemia development (n = 5-7). Ctrl, Control mice [11]. (D–E). Absolute numbers of CD45.1+LKS+ (D) and CD45.1+LKS− (E) cells in leukemic BM (n = 4-5) [11]. (PNG 71 kb) [file 12918_2016_308_MOESM1_ESM.png]

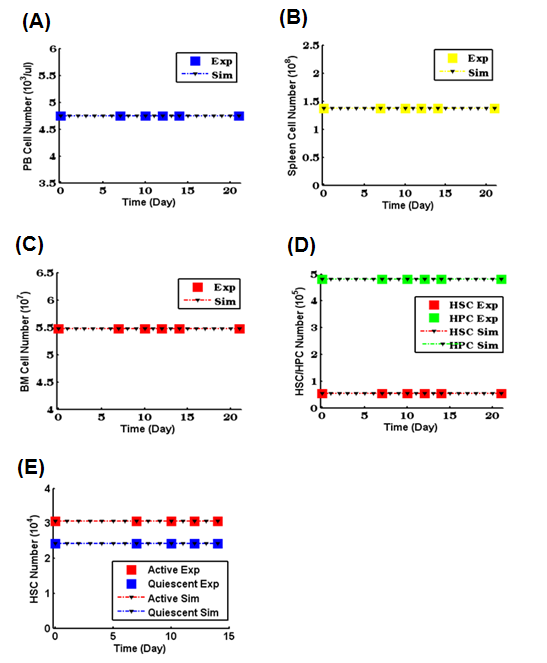

Supplement: Additional file 2: Figure S2. — Reproduction of the normal control by the model. (A–C). Computational and experimental cell kinetics of PB (A), spleen (B) and BM (C) under the normal condition. The computation results are yielded by directly eliminating the parameters for leukemic effects in the mathematical model. Experimental data are taken from Ref [11]. (D). Computational and experimental kinetics of BM HSCs/HPCs under the normal condition. (E). Computational and experimental kinetics of quiescent and active HSCs in BM under the normal condition. (PNG 79 kb) [file 12918_2016_308_MOESM2_ESM.png]

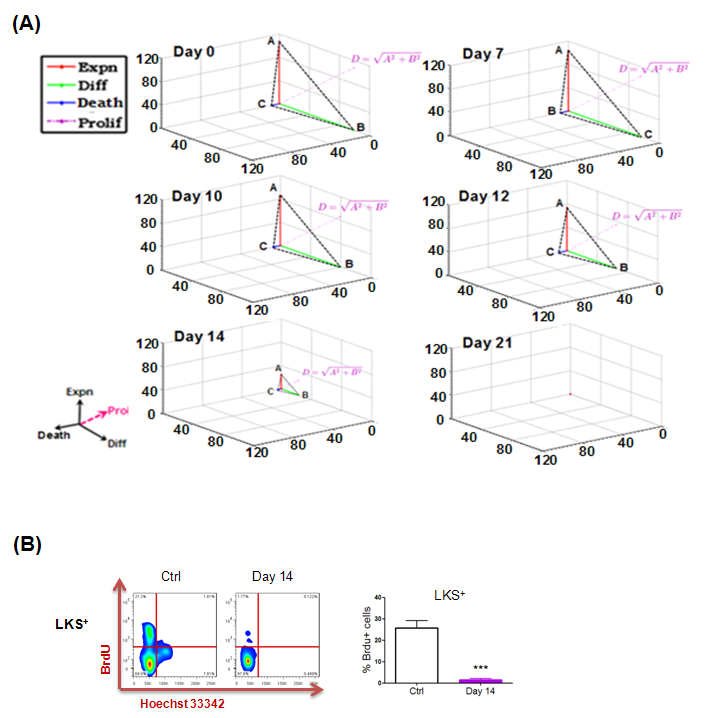

Supplement: Additional file 3: Figure S3. — Supplemental data for illustrating the major factor of HSC loss. (A). The 3D visualization of projections of the HSC dynamics. Expn, Diff and Death are the orthogonal axes; and the rates projected on them are bars with lengths proportional to the values. Prolif is symbolized as the vectorized composition of Diff and Expn. Temporal profiles at day 0, 7, 10, 12, 14 and 21 are given. (B). Flow plots (left panel) and histogram (right panel) show the BrdU incorporation of HSCs (LKS+ cells) in leukemic BM. Data are represented as the mean ± SEM (n = 8, 2 independent experiments). *** p < 0.001 [11]. (PNG 165 kb) [file 12918_2016_308_MOESM3_ESM.png]

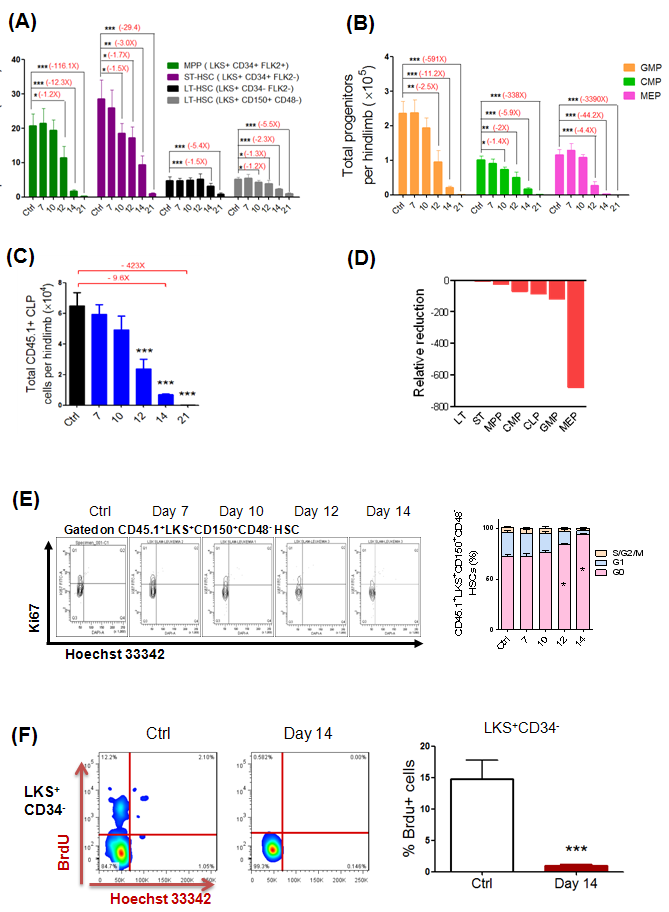

Supplement: Additional file 4: Figure S4. — Supplemental data for illustrating the differentiation blockade in the hematopoietic cascade. (A). Absolute numbers of LT-HSCs, ST-HSCs and MPPs in leukemic BM [11]. (B–C). Absolute numbers of CMPs, GMPs, MEPs (B) and CLPs (C) in leukemic BM. Data are represented as the mean ± SEM (n = 12, 3 independent experiments). * p < 0.05, ** p < 0.01, *** p < 0.001. + or -, increase or decrease [11]. (D). A pattern showing linear correlation between the reduction and differentiation hierarchy in the hematopoietic cascade. The reduction of LT-HSC was normalized to −1, and the bars indicate normalized reduction level [11]. (E). Flow plots (left panel) and histograms (right panel) show the cell cycle status of LT-HSCs in leukemic BM. Data are represented as the mean ± SEM (n = 12, 3 independent experiments). * p < 0.05 [11]. (F). Flow plots (left panel) and histogram (right panel) show the BrdU incorporation of LT-HSCs in leukemic BM. Data are represented as the mean ± SEM (n = 8, 2 independent experiments). *** p < 0.001 [11]. (PNG 135 kb) [file 12918_2016_308_MOESM4_ESM.png]

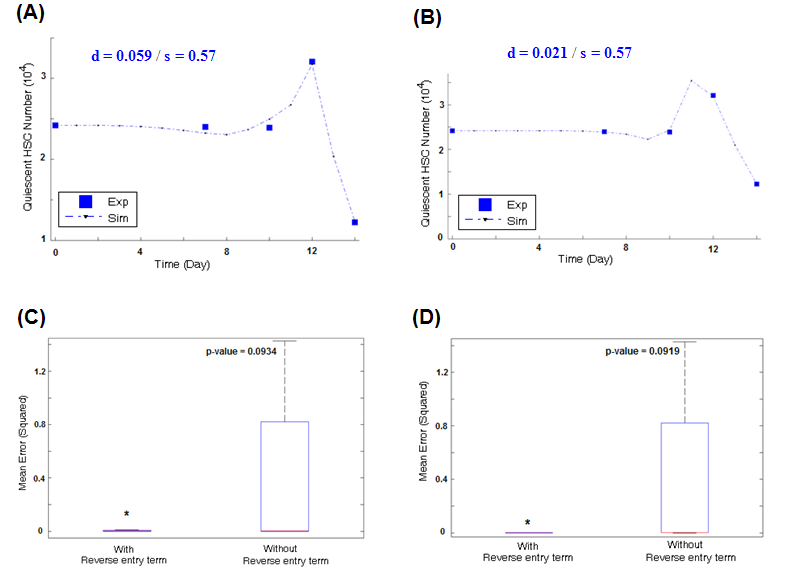

Supplement: Additional file 5: Figure S5. — Supplemental data for illustrating the increased quiescence of HSCs. (A-B). Fitness of the models including the G0 re-entry, which adopts two other forms: the Hill form (A) and exponential form (B). Reference data for the kinetics of quiescent HSCs were taken from the cell cycle analyses (Fig. 3c) [11], in which percentage numbers (G0) were transformed into absolute numbers by multiplying the total cell count of HSC population (CD45.1+LKS+, Additional file 1: Figure S1D). (C–D). Box plots show that the mean errors of the models with G0 re-entry are uniformly lower than the plain model (one-tail t-test, * p < 0.1). (PNG 54 kb) [file 12918_2016_308_MOESM5_ESM.png]
