# Supplementary material for: Digitalization of a non-irradiated acute myeloid leukemia model
Source: BMC Syst Biol. 2016 Aug 26;10(Suppl 3):64. doi: 10.1186/s12918-016-0308-x (PMC5009825; doi:10.1186/s12918-016-0308-x)
Supplement: Additional file 6: — Descriptions in-detail for the computational model. Descriptions of the modeling process and computational procedures are enclosed; concrete mathematical formulas and (optimized) parameter values are also provided. (DOCX 152 kb) [file 12918_2016_308_MOESM6_ESM.docx]

Text S1

**Supplemental materials for computational modeling**

Enclosed herein are the modeling assumptions/conceptions, computational procedures, and descriptions of equations/kinetic parameters.

**Dynamics of (Total) Hematopoietic Cells in PB:**

We assume that spleen and BM are two compartments and PB is the medium running through them, thus direct connections BM ←→ PB and spleen ←→ PB are defined and the inter-connection between BM and spleen is via PB. In PB, as most of hematopoietic cells are mature, we neglect their proliferative abilities. Thus, hematopoietic cells have only three actions, import, export (PB circulation) and death. Leukemia cells can proliferate and migrate in the circulation, and their death is not considered because of their tremendous growth advantage. Because the PB also connects with other tissues in addition to spleen and BM, it actually exhibits an overall effect of all cells’ actions. Therefore, PB is modeled independently of spleen and BM in terms of the average net-effect of all actions relating to it. Thus, the effect of overall import action can be simplified as a constant *A*, and we can merge the effects of cell death and export as the overall decay of PB cells. Hence, the dynamics of normal cells (*N*) in the PB is given by

$$\frac{dN}{dt}=A-f_{Decay}(N)$$

Likewise, the dynamics of leukemia cells (*L*) in PB can be given by

$$\frac{dL}{dt}=f_{Prolif}(L)+B$$

where *f_Prolif_* (*L*) encodes the proliferation of *L*, and the overall import/export effects are merged into one parameter *B* because they are both constants (if export > import, *B* < 0; and otherwise, *B* > 0). Both the *f_Decay_*(*N*) and *f_Prolif_* (*L*) take appropriate kinetic forms but are not likely to be zero^th^-order, as the corresponding processes are related to *N* and *L*. Under leukemia, *N* is influenced by *L*, while *N* exerts little influence on *L*; thus Eq. S3 must be modified with the functional influences of *L*, and thus the dynamics of *N* takes the form:

$$\frac{dN}{dt}=A\cdot K_{1}(L)-f_{Decay}(N,K_{2}(L))$$

where *K_1_* and *K_2_* are the functions encoding the functional influences.

**Dynamics of (Total) Hematopoietic Cells in Spleen or BM:**

In hematopoietic tissues (spleen and BM), normal cells have an additional action of proliferation because primitive hematopoietic cells (HSCs/HPCs) exist. Meanwhile, hematopoietic tissues export cells as one of their functions, and this action is different in nature and mechanism from PB circulation. Again, leukemic cells enter the tissues, proliferate in them or move out of them, and deaths of leukemic cells were neglected because they possess large growth advantages over normal cells. Cell dynamics in spleen or BM are modeled precisely. For normal cells (*N*), the dynamics involve all actions, proliferation, death, export and import (i.e. cell mobility due to PB circulation). Because the export and import are different in nature and mechanism in this case, the corresponding terms cannot be merged. Therefore, the dynamics of normal cells are given by:

$$\frac{dN}{dt}=f_{Prolif}(N,K_{1}(L))-f_{Death}(N,K_{2}(L))-f_{Export}(N,K_{3}(L))+f_{Import}$$

where *K_1, 2, 3_* encode the functional influences of leukemic cells (*L*). Similarly, the dynamics of leukemia cells is expressed as:

$$\frac{dL}{dt}=f_{Prolif}(L)-f_{Export}(L)+f_{Import}$$

The function for cell migration into hematopoietic tissues, *f_Import_*, encodes the rate at which cells in PB reflux into the tissues. We explicitly consider the effects to be related with the numbers of *L* and *N* in PB; and the simplest way is assuming the simple gradient migration/diffusion of particles, which stipulates that the potential for spontaneous movement of particles from their current location to somewhere else depends on their relative concentration. Thus the functions adopt the forms of *φ*_0_ · (*L* /*L*+*N*) and *ψ*_0_·(*N* /*L*+*N*) for the refluxes of *L* and *N,* respectively, where *φ_0_* and *ψ_0_* are the efficiency coefficients depending on properties of both the cells themselves (e.g., the treacliness, shape, etc.) and the vessels/channels of hematopoietic tissues in which blood cells move.

**Dynamics of HSCs/HPCs:**

HSCs are capable of undergoing self-renewal (expansion), differentiation and death, whereas HPCs cannot self-renew and their number is reinforced by the differentiation of the upstream HSCs. Meanwhile, we stipulate that the HSCs/HPCs are mainly located in hematopoietic tissues (e.g. BM), as the quantities of primitive cells in PB are quite small. For HSCs (*S*), proliferation is considered to be two processes, namely expansion (*Expn*) and differentiation (*Diff*), while export and import are neglected. For HPCs (*P*), proliferation is only differentiation. Therefore, their dynamics (with the functional influences of *L*) are interpreted by the following equations:

$$\left\{\begin{matrix} \frac{dS}{dt}=f_{Expn}(S,K_{1}(L))-f_{Diff}(S,K_{2}(L))-f_{Death}(S,K_{3}(L)) \\ \frac{dP}{dt}=f_{Diff}(S,K_{2}(L))-f_{Diff}(P,K_{4}(L))-f_{Death}(P,K_{5}(L)) \end{matrix}\right.$$

**Kinetic Rate Forms for Cell Actions:**

The rates of cell actions may follow any of the possible kinetic forms, namely, the zeroth-order kinetics, first-order kinetics, exponential kinetics, and the Hill/Michealis-Menten kinetics, etc., which have physical and biological implications and are commonly used to model complex bio-processes ^1-3^. The functional influences of leukemic cells can be modeled as the Hill form, in which the acting effects are encoded by the saturation constant *θ* and the order of action *n* as *θ^n^ /*(*L^n^+θ^n^*) for inhibitory effects ^3,4^.

In the model framework described by Eq. S2-S6, *A* and *B* are constants; elsewhere, the function forms$f_{Prolif},f_{Death},f_{Export}\in\left\{\alpha,\beta x,\beta x^{n},\beta x/\left(x^{n}+\theta^{n}\right)\right\}^{*}$.and $K_{i}=\theta_{i}^{n_{i}}/\left(x^{n_{i}}+\theta_{i}^{n_{i}}\right)$apply.

**Numerical Optimization:**

The canonical form of parameter optimization is shown as Eq. S7. The reference data used in the optimization were the experimental cell kinetics only. We used the Genetic Algorithm (GA) as the principal approach for *de novo* parameter optimization in the pool of dynamic equations of different mathematical forms.

$$\begin{matrix} \text{min }S(\mu)=\sum_{i=1}^{m} \left[y_{i}-x_{i}(t_{i},\vec{\mu})\right]^{2} \\ \text{s.t. }\frac{d\vec{x}}{dt}=f(\vec{x},t,\vec{\mu}) \\ \text{where }\left\{\begin{matrix} \vec{y}=(y_{1},...,y_{m})^{T}\in D \\ \vec{x}=(x_{1}(t),...,x_{n}(t))^{T},t=t_{1},t_{2},...,t_{m} \\ \vec{\mu}=(\mu_{1},...,\mu_{p})^{T}\in R_{+}^{p} \\ f:R_{+}^{n}\times R_{+}\times R_{+}^{p}\to R_{+}^{n},f\in C \end{matrix}\right. \end{matrix}$$

where *C* is the collection of formulas as discussed earlier, and *D* is our reference dataset (experimental). We also used other methods such as the trust-region method, interior-point method, active-set method, etc. along with GA to enhance the efficiency of computation. The numerical criterion is that the mean deviation of the computed values (defined as the root-mean-square deviation, *d*) is no larger than the experimental standard deviation (*s*), i.e., *d* ≤ *s*.

**Mathematical Model:**

Detailed formulas for the dynamical equation system are provided below. For detailed descriptions of symbols and annotations, and values of parameters, see the lists appended at the end of this document (Table A, B).

**^*^*Notes*: ^i^ Simulations/Predictions on cell kinetics can be obtained by solving the above dynamic equations with respect to certain initial values. ^ii^ The blue typed parts denote the effects of leukemia. If we remove these parts from the model directly (without changing anything else), the residual parts naturally represent the kinetics under normal condition (Figure S2).**

**Model Selection**

We evaluated the importance of cell mobility to the modeling of multi-tissue hematopoiesis, and we also analyzed the potential existence of a mechanism that could bring HSCs into the G0 phase (under leukemia) using *in silico* model selection. Different models can be formulated according to whether a hypothesis or its null counterpart is adopted. Statistical tests are then performed to determine which models are more likely to produce the experimental data by comparing their mean errors in model fitting (one-tail *t*-test).

***Cell mobility:***

Equations for cell kinetics in hematopoietic tissues with cell mobility (i.e., migrations or refluxes of cells from PB into spleen/BM) included are shown in Eq. S8-S13. Intuitively, the counterpart model neglecting cell mobility is formulated by disregarding the effects or keeping them constant independent of the cell kinetics in PB (Eq. S16).

$$\left\{\begin{matrix} \frac{dL}{dt}=f_{Prolif}(L)-f_{Export}(L)+\alpha\\ \frac{dN}{dt}=f_{Prolif}(N,K_{1}(L))-f_{Death}(N,K_{2}(L))-f_{Export}(N,K_{3}(L))+\beta\\ \alpha,\beta\in\left\{0\right\}\cup R_{+} \end{matrix}\right.$$

Numerical performances of the models representing the two scenarios were compared in terms of the mean errors when fitting to the real data. The comparison was performed using the one-tailed *t*-test to examine if the fitness of one model was significantly lower than that of the other.

***G0 re-entry:***

Active and quiescent HSCs have inter-changes via certain cellular processes; in general, some active HSCs directly generate quiescent daughter cells in proliferation, which is the input for quiescent HSCs. A portion of quiescent HSCs spontaneously enter the cell cycle, supplementing the active HSCs and thus consolidating the hematopoietic pool. Under normal conditions, HSCs that already entered the cell cycle tend to sustain their functions and are unlikely to return to a quiescent state; therefore, we regard the spontaneous effect of aHSC -> qHSC to be very small ^5,6^. We intended to numerically illustrate that under leukemia, there is likely to be a strengthened reverse action bringing more HSCs back into the G0 phase so that the experimental cell cycle kinetics of HSCs can be explained. The dynamic equations of active (a) and quiescent (q) HSCs (Eq. S17-S18) were derived by sub-dividing the HSC equation (Eq. S14) in accordance to the two sub-populations of HSCs. Eq. S17 and S18 represent two different scenarios in which the reverse entry term does or does not exist. Literally, as maintenance of logical integrity, the theoretical sum of the respective (a) and (q) equations in either Eq. S17 or S18 equals Eq. S14 (i.e. the overall HSC dynamics).

*Without G0 re-entry*:

$$\left\{\begin{matrix} \frac{d[a]}{dt}=f_{Prolif,a\to a}([a],\tilde{K}_{1}(L))+f_{Cycling}([q],\tilde{K}_{2}(L))-f_{Death}([a],\tilde{K}_{3}(L))-f_{Diff}([a],\tilde{K}_{4}(L)) \\ \frac{d[q]}{dt}=f_{Prolif,a\to q}([a],\tilde{K}_{1}(L))-f_{Cycling}([q],\tilde{K}_{2}(L)) \end{matrix}\right.$$

where the subscript “a→a” means active HSCs (a) proliferating into active HSCs (a); “a→q” means active HSCs (a) generating quiescent offspring HSCs (q); *f_Cycling_* stands for the rate that quiescent HSCs (q) spontaneously enter the cell cycle (under the normal condition); and *f_Death_* and *f_Diff_* are the death and differentiation rates of active HSCs. The forms of the functional influences of *L* (i.e. *K*_1,2,3,4_) resemble those in the earlier contexts.

*With G0 re-entry:*

$$\left\{\begin{matrix} \frac{d[a]}{dt}=f_{Prolif,a\to a}([a],\tilde{K}_{1}(L))+f_{Cycling}([q],\tilde{K}_{2}(L))-f_{Death}([a],\tilde{K}_{3}(L))-f_{Diff}([a],\tilde{K}_{4}(L))-g([a],L) \\ \frac{d[q]}{dt}=f_{Prolif,a\to q}([a],\tilde{K}_{1}(L))-f_{Cycling}([q],\tilde{K}_{2}(L))+g([a],L) \end{matrix}\right.$$

where the appended function *g*([*a*],*L*) (red color) represents the rate of G0 re-entry.

Theoretically, *g*([*a*],*L*) can be any reasonable form, such as a polynomial, fraction, etc. Without losing generality, we allowed the function to traverse a variety of possible forms and showed the results using the three most common forms, namely, the linear form (*g_1_*), the Hill form (*g_2_*), and the exponential form (*g_3_*). Our aim is to determine if all (or a subset) of the equations with G0 re-entry could describe the experimental data better than the equations without the function *g*([*a*],*L*), so that the rationale for the existence of G0 re-entry could be supported.

$g_{1}(x,L)=g\cdot\left(\frac{L^{n}}{L^{n}+\theta^{n}}\right)\cdot x$;

$g_{2}(x,L)=\frac{gx^{n}}{\theta^{n}\left(1+K_{a}/L\right)^{n}+x^{n}}$;

$$g_{3}(x,L)=g\cdot\left(\frac{L^{n}}{L^{n}+\theta^{n}}\right)\cdot x^{m}$$

The concrete forms of the dynamics of active (a) and quiescent (q) HSCs without and with G0 re-entry are shown in Eq. S19 and S20, respectively, with descriptions for the equation parameters listed in Table C. Numerical methods stated in previous contexts are utilized during computation, and statistical tests are implemented to compare the fitness degrees (of Eq. S19 and S20) to real kinetics.

Again, the blue-colored sections can be removed from Eq. S19 and S20 (without anything else being changed) to reflect the kinetics under normal conditions (see Figure S2E); in fact, they can be reduced to the same form in the absence of leukemia. Refer to the parameter list (Table C) for detailed information.

**Table A: Variables.**

| **Denotation** | **Description** | **Unit** |
| --- | --- | --- |
| [*L_PB_*] | Leukemia cell number in the peripheral blood. | *10^3^ul^-1^* |
| [*N_PB_*] | Normal cell number in the peripheral blood. | *10^3^ul^-1^* |
| [*L_S_*] | Leukemia cell number in spleen. | *10^8^* |
| [*N_S_*] | Normal cell number in spleen. | *10^8^* |
| [*L_BM_*] | Leukemia cell numbers in the bone marrow. | *10^7^* |
| [*N_BM_*] | Normal cell numbers in the bone marrow. | *10^7^* |
| [*HSC_BM_*] | HSC numbers in the bone marrow. | *10^4^* |
| [*HPC_BM_*] | HPC numbers in the bone marrow. | *10^5^* |

**Table B: Parameters.**

| **Symbol** | **Description** | **Value** | **Unit** |
| --- | --- | --- | --- |
| *k_1_* | the overall proliferation rate scalar of leukemia cells in PB; quasi first-order kinetics | 0.5399 | *Day^-1^* |
| *A* | the overall influx rate of normal cells into PB from other hematopoietic organs/tissues | 6.069 | *10^3^μl^-1^Day^-1^* |
| *f_0_* | the action efficiency scalar of leukemia cells on the overall influx rate of normal cells in PB | 1.8694 | metrics free |
| *κ_1_* | the number level of leukemia cells leading to 50% attenuation of the overall influx rate of normal cells in PB | 0.9102 | *10^3^μl^-1^* |
| *s_1_* | the Hill index of the inhibitory effect of leukemia cells on the overall influx rate of normal cells in PB | 50.8072 | metrics free |
| *β_1_* | the overall decay rate scalar of normal cells in PB; quasi first-order kinetics, lumping together the apoptosis of cells and the outflux of cells from PB into other organs/tissues | 1.2777 | *Day^-1^* |
| *k_0_* | similar to *f_0_* | 1.2031 | metrics free |
| *κ_2_* | similar to *κ_1_* | 0.5007 | *10^3^μl^-1^* |
| *s_2_* | similar to *s_1_* | 7.4456 | metrics free |
| *β* | the maximum proliferation rate scalar of leukemia cells in spleen; Hill kinetics | 7.8850 | *10^8^Day^-1^* |
| *n* | the Hill index of the leukemia cells growth kinetics in spleen | 1.4656 | metrics free |
| *θ* | the number level of leukemia cells leading to 50% of the maximum growth rate of leukemia cells in spleen | 5.7987 | *10^8^* |
| *γ* | the maximum export rate scalar of leukemia cells from spleen; Hill kinetics | 11.0225 | *10^8^Day^-1^* |
| *m* | the Hill index of the leukemia cells export rate from spleen | 7.9782 | metrics free |
| *η* | the number level of leukemia cells leading to 50% of the maximum export rate of leukemia cells from spleen | 5.5541 | *10^8^* |
| *γ1* | the export rate scalar of normal cells from spleen; quasi first-order kinetics | 1.0565 | *Day^-1^* |
| *γ2* | the apoptosis rate scalar of normal cells in spleen; quasi-first-order kinetics | 13.8169 | *Day^-1^* |
| *γ3* | the proliferation rate scalar of normal cells in spleen; quasi first-order kinetics | 13.7617 | *Day^-1^* |
| *θ_1_* | the number level of leukemia cells leading to 50% attenuation of the regular export rate of normal cells from spleen | 4.4609 | *10^8^* |
| *θ_2_* | the number level of leukemia cells leading to 50% increment of the regular apoptosis rate of normal cells in spleen | 26.3004 | *10^8^* |
| *θ_3_* | the number level of leukemia cells leading to 50% attenuation of the regular proliferation rate of normal cells in spleen | 21.6686 | *10^8^* |
| *n_1_* | the Hill index of the inhibitory effect of leukemia cells on the export rate of normal cells from spleen | 0.4262 | metrics free |
| *n_2_* | the Hill index of the acceleration effect of leukemia cells on the apoptosis rate of normal cells in spleen | 26.8721 | metrics free |
| *n_3_* | the Hill index of the inhibitory effect of leukemia cells on the proliferation rate of normal cells in spleen | 27.2880 | metrics free |
| *f_2_* | the effect scalar of leukemia cells on the apoptosis rate of normal cells in spleen | 10.4598 | metrics free |
| *Φ_0_* | the proportional cell reflux rate scalar of leukemia cells back into spleen from PB | 1.0184 | *10^8^Day^-1^* |
| *Ψ_0_* | the proportional cell reflux rate scalar of normal cells back into spleen from PB | 1.5230 | *10^8^Day^-1^* |
| *ρ* | the maximum proliferation rate scalar of leukemia cells in BM; Hill kinetics | 3.4270 | *10^7^Day^-1^* |
| *r* | the Hill index of the leukemia cells growth kinetics in BM | 3.9519 | metrics free |
| *λ* | the number level of leukemia cells leading to 50% of the maximum growth rate of leukemia cells in BM | 1.8680 | *10^7^* |
| *δ* | the maximum export rate scalar of leukemia cells from BM; Hill kinetics | 12.1311 | *10^7^Day^-1^* |
| *σ* | the number level of leukemia cells leading to 50% of the maximum export rate of leukemia cells from BM | 3.7982 | *10^7^* |
| *k* | the Hill index of the leukemia cells export rate of BM | 12.9417 | metrics free |
| *ρ_1_* | the export rate scalar of normal cells from BM; quasi first-order kinetics | 1.2015 | *Day^-1^* |
| *ρ_2_* | the apoptosis rate scalar of normal cells in BM; quasi first-order kinetics | 1.1512 | *Day^-1^* |
| *ρ_3_* | the proliferation rate scalar of normal cells in BM; quasi first-order kinetics | 2.2875 | *Day^-1^* |
| *λ_1_* | the number level of leukemia cells leading to 50% attenuation of the regular export rate of normal cells from BM | 4.3655 | *10^7^* |
| *λ_2_* | the number level of leukemia cells leading to 50% increment of the regular apoptosis rate of normal cells in BM | 4.1258 | *10^7^* |
| *λ_3_* | the number level of leukemia cells leading to 50% attenuation of the regular proliferation rate of normal cells in BM | 6.2345 | *10^7^* |
| *r_1_* | the Hill index of the inhibitory effect of leukemia cells on the export rate of normal cells from BM | 1.8900 | metrics free |
| *r_2_* | the Hill index of the acceleration effect of leukemia cells on the apoptosis rate of normal cells in BM | 1.4219 | metrics free |
| *r_3_* | the Hill index of the inhibitory effect of leukemia cells on the proliferation rate of normal cells in BM | 7.0574 | metrics free |
| *g_2_* | similar to *f_2_* | 2.7310 | metrics free |
| *Ω_0_* | the proportional cell reflux rate scalar of leukemia cells back into BM from PB | 5.0559 | *10^7^Day^-1^* |
| *Θ_0_* | the proportional cell reflux rate scalar of normal cells back into BM from PB | 0.3569 | *10^7^Day^-1^* |
| *k_1_’* | the proliferation rate scalar of HSCs in BM; quasi first-order kinetics | 20.3859 | *Day^-1^* |
| *η_1_* | the number level of leukemia cells leading to 50% attenuation of the regular proliferation rate of HSCs in BM | 4.5947 | *10^4^* |
| *m_1_* | the Hill index of the inhibitory effect of leukemia cells on the proliferation rate of HSCs in BM | 9.4659 | metrics free |
| *γ_1_’* | the apoptosis rate scalar of HSCs in BM; quasi first-order kinetics | 1.5156 | *Day^-1^* |
| *f_2_’* | similar to *f_2_* | 2.2120 | metrics free |
| *η_2_* | the number level of leukemia cells leading to 50% increment of the regular apoptosis rate of HSCs in BM | 9.0133 | *10^4^* |
| *m_2_* | the Hill index of the acceleration effect of leukemia cells on the apoptosis rate of HSCs in BM | 1.1507 | metrics free |
| *ρ_1_’* | the differentiation rate scalar of HSCs to HPCs in BM; quasi first-order kinetics | 18.8703 | *Day^-1^* |
| *η_3_* | the number level of leukemia cells leading to 50% attenuation of the regular differentiation rate of HSCs in BM | 8.8752 | *10^4^* |
| *m_3_* | the Hill index of the inhibitory effect of leukemia cells on the differentiation rate of HSCs in BM | 2.3473 | metrics free |
| *ρ_2_’* | the differentiation rate scalar of HPCs to downstream cell types in BM; exponential kinetics | 1.5499 | *Day^-1^* |
| *η_4_* | the number level of leukemia cells leading to 50% attenuation of the regular differentiation rate of HPCs in BM | 6.2134 | *10^5^* |
| *n_4_* | the Hill index of the inhibitory effect of leukemia cells on the differentiation rate of HPCs in BM | 0.4354 | metrics free |
| *δ’* | the apoptosis rate scalar of HPCs in BM; quasi first-order kinetics | 0.5221 | *Day^-1^* |
| *f_3_* | similar to *f_2_’* | 17.9646 | metrics free |
| *η_5_* | the number level of leukemia cells leading to 50% increment of the regular apoptosis rate of HPCs in BM | 5.6387 | *10^5^* |
| *n_5_* | the Hill index of the acceleration effect of leukemia cells on the apoptosis rate of HPCs in BM | 1.5660 | metrics free |

**Table C: Parameters in the modeling of active/quiescent HSCs.**

| **Symbol** | **Description** | ***g_1_*** | ***g_2_*** | ***g_3_*** | **Unit** |
| --- | --- | --- | --- | --- | --- |
| $\tilde{\theta}_{1}$ | the number of leukemia cells leading to 50% attenuation of the regular proliferation rate of active HSC | 4.1112 | 3.0629 | 1.4411 | *10^4^* |
| $\tilde{\theta}_{2}$ | the number of leukemia cells leading to 50% attenuation of the regular rate of quiescent HSC to spontaneously enter cell cycle | 2.0383 | 1.5932 | 6.6232 | *10^4^* |
| $\tilde{\theta}_{3}$ | the number of leukemia cells leading to 50% increment of the regular apoptosis rate of active HSC | 11.2519 | 4.2513 | 4.4028 | *10^4^* |
| $\tilde{\theta}_{4}$ | the number of leukemia cells leading to 50% attenuation of the regular differentiation rate of active HSC | 11.0910 | 5.4801 | 0.6821 | *10^4^* |
| $\tilde{n}_{1}$ | the Hill index of the inhibitory effect of leukemia cells on the proliferation rate of active HSC | 12.0715 | 9.9738 | 1.6584 | metrics free |
| $\tilde{n}_{2}$ | the Hill index of the inhibitory effect of leukemia cells on the rate of quiescent HSC to spontaneously enter cell cycle | 1.6332 | 1.9671 | 7.7915 | metrics free |
| $\tilde{n}_{3}$ | the Hill index of the acceleration effect of leukemia cells on the apoptosis rate of active HSC | 1.3423 | 0.8813 | 23.2898 | metrics free |
| $\tilde{n}_{4}$ | the Hill index of the inhibitory effect of leukemia cells on the differentiation rate of active HSC | 1.8717 | 4.4436 | 154.2495 | metrics free |
| $\tilde{k}$ | the proliferation rate scalar of active HSC; quasi first-order kinetics | 35.8374 | 5.1227 | 1.4977 | *Day^-1^* |
| $\tilde{\delta}$ | the proportional effect coefficient of the direct generation of quiescent HSC from the proliferation of active HSC | 10.7747 | 2.7122 | 0.4373 | *Day^-1^* |
| *c* | the spontaneous cell cycle entry rate scalar of quiescent HSC; quasi first-order kinetics | 13.6134 | 3.4267 | 0.5525 | *Day^-1^* |
| $\tilde{\gamma}$ | the apoptosis rate scalar of active HSC; quasi first-order kinetics | 2.0954 | 2.3303 | 1.2787 | *Day^-1^* |
| *f_3_’* | similar to *f_2_’* | 8.5273 | 0.4440 | 435.5445 | metrics free |
| *d* | the differentiation rate scalar of active HSC to downstream cell types; quasi first-order kinetics | 33.7420 | 2.7924 | 0.2190 | *Day^-1^* |

**^*^Notes: Parameter values are shown for the respective equations in which the functions *g_1_*, *g_2_* and *g_3_* were adopted.**

**Supplemental References**

1. Savageau, M.A. & Voit, E.O. Recasting nonlinear differential equations as S-systems: a canonical nonlinear form. *Math Biosci* **87**, 83-115 (1987).

2. Voit, E.O. *Computational analysis of biochemical systems*, (Cambridge University Press, Cambridge, 2000).

3. Sorribas, A., Hernández-Bermejo, B., Vilaprinyo, E. & Alves, R. Cooperativity and saturation in biochemical networks: A saturable formalism using Taylor series approximations. *Biotechnol Bioeng* **97**, 1259-1277 (2007).

4. Colijn, C. & Mackey, M.C. A mathematical model of hematopoiesis - I. Periodic chronic myelogenous leukemia. *J Theor Biol* **237**, 117-132 (2005).

5. Macarthur, B.D., Ma'ayan, A. & Lemischka, I.R. Systems biology of stem cell fate and cellular reprogramming. *Nat Rev Mol Cell Biol* **10**, 672-681 (2009).

6. Wang, J., Xu, L., Wang, E. & Huang, S. The potential landscape of genetic circuits imposes the arrow of time in stem cell differentiation. *Biophysical journal* **99**, 29-39 (2010).
